# Integrating Clinical Data and Attentional CT Imaging Features for Esophageal Fistula Prediction in Esophageal Cancer

**DOI:** 10.3389/fonc.2021.688706

**Published:** 2021-11-23

**Authors:** Yiyue Xu, Hui Cui, Taotao Dong, Bing Zou, Bingjie Fan, Wanlong Li, Shijiang Wang, Xindong Sun, Jinming Yu, Linlin Wang

**Affiliations:** ^1^ Department of Radiation Oncology, Shandong Cancer Hospital and Institute, Shandong First Medical University and Shandong Academy of Medical Sciences, Jinan, China; ^2^ Tianjin Medical University Cancer Institute and Hospital, National Clinical Research Center for Cancer, Key Laboratory of Cancer Prevention and Therapy, Tianjin, China; ^3^ Department of Oncology, Tianjin Medical University, Tianjin, China; ^4^ Department of Computer Science and Information Technology, La Trobe University, Melbourne, VIC, Australia; ^5^ Department of Obstetrics and Gynecology, Qilu Hospital of Shandong University, Jinan, China

**Keywords:** esophageal cancer, esophageal fistula, radiomics, deep learning, prediction model

## Abstract

**Background and Purpose:**

This study aims to develop a risk model to predict esophageal fistula in esophageal cancer (EC) patients by learning from both clinical data and computerized tomography (CT) radiomic features.

**Materials and Methods:**

In this retrospective study, computerized tomography (CT) images and clinical data of 186 esophageal fistula patients and 372 controls (1:2 matched by the diagnosis time of EC, sex, marriage, and race) were collected. All patients had esophageal cancer and did not receive esophageal surgery. 70% patients were assigned into training set randomly and 30% into validation set. We firstly use a novel attentional convolutional neural network for radiographic descriptor extraction from nine views of planes of contextual CT, segmented tumor and neighboring structures. Then clinical factors including general, diagnostic, pathologic, therapeutic and hematological parameters are fed into neural network for high-level latent representation. The radiographic descriptors and latent clinical factor representations are finally associated by a fully connected layer for patient level risk prediction using SoftMax classifier.

**Results:**

512 deep radiographic features and 32 clinical features were extracted. The integrative deep learning model achieved C-index of 0.901, sensitivity of 0.835, and specificity of 0.918 on validation set with superior performance than non-integrative model using CT imaging alone (C-index = 0.857) or clinical data alone (C-index = 0.780).

**Conclusion:**

The integration of radiomic descriptors from CT and clinical data significantly improved the esophageal fistula prediction. We suggest that this model has the potential to support individualized stratification and treatment planning for EC patients.

## Introduction

EC is the 8th most common tumor worldwide (1), and nearly half of the cases are found in China (2). Patients with EC achieve improved prognosis with recent advance in radiotherapy, chemotherapy and immunotherapy (3). However, the treatment outcome of patients who developed esophageal fistula, a severe complication of EC, is still well below satisfaction and expectation. Perforation may lead to prolonged infection, poor nutrition, sepsis, and even massive hemorrhage, which can considerably affect survival. It is reported that the median post-fistula survival time of EC patients with esophageal fistula was approximately 3.63 months (4). Therefore, predicting esophageal fistula before treatment is highly desirable to improve prognosis in EC patients.

Previous studies on esophageal fistula mostly focused on clinical parameters, using the logistics regression analysis to establish predictive models (5–7). Such researches cannot effectively handle the complex relationship between esophageal fistula and numerous risk factors in the real world, and the predictive efficacy cannot meet the needs. Moreover, the importance of CT imaging has never been reported. The radiographic features contained in CT images, such as tumor texture features, tumor size, and other morphological information, are important potential biomarker (8). Previous research reported its application in predicting the survival (8), lymph node metastasis (9) and treatment response (10) in EC patients. Combining CT imaging and clinical features can more accurately predict esophageal fistula.

Deep learning methods can identify non-linear relationships between different types of parameters, and have been explored in large data analysis (11) and medical images diagnosis (12). However, there is no deep learning study involving esophageal fistula.

In this study, we developed a deep learning model of esophageal fistula for EC patients. Our model automatically extracted the information in the CT imaging and integrated the clinical features. In addition, the attention map was drawn to visualize the neural network based on CT images.

## Materials and Methods

### Patients

This retrospective study was approved by the local review board. For this type of study, formal informed consent was not required, and all collected data was kept confidential and anonymous. EC patients who developed esophageal fistula in Shandong Cancer Hospital from July 2014 to August 2019 were retrospectively enrolled as the case group. Patients who were clearly described with esophageal fistula or perforation in CT, esophagogram or endoscopy systems were collected. Because anastomotic fistula is a special type of esophageal fistula closely related to surgical methods and surgical techniques, our study did not involve anastomotic fistula after esophagus surgery. We only study the esophageal fistula caused by tumor itself and treatment. The inclusion criteria included: 1) patients diagnosed as EC pathologically with the World Health Organization (WHO) criteria; 2) availability of general, diagnostic and therapeutic data; 3) availability of contrast-enhanced CT imaging before treatment; 4) diagnosed as esophageal fistula by either endoscopy, CT or contrast radiography of the upper gastrointestinal tract. Exclusion criteria were: 1) patients treated by esophageal surgery; 2) the fistula developed due to medical injure or trauma; 3) concomitant with another carcinoma. By such, there are 186 eligible patients. At the same time, we collected a control group of 372 patients, 1:2 matched with the case group by the diagnosis time of EC, sex, marriage, and race. Patients in the control group followed the same inclusion and exclusion criteria as above but didn’t develop esophageal fistula. The included patients were divided into training set (n = 390) and validation set (n = 168) randomly. Specifically, We applied the method of simple randomization to separate the whole dataset into training and validation sets using random numbers generated by the computer.

### Clinical Data Collection

We collected data from medical records using a standardized questionnaire about general, diagnostic, therapeutic and esophageal fistula data. Specifically, general parameters include gender, age at initial diagnosis, Eastern Cooperative Oncology Group performance status (ECOG PS) score, Body Mass Index (BMI), history of smoking, history of drinking, history of hypertension, history of diabetes, history of coronary heart disease and eating obstruction. Diagnostic parameters include tumor stage (T4), node stage (N2-3), stage, tumor site, longitudinal length of lesions, pathological and general type. Therapeutic parameters consist of chemotherapy, radiotherapy, target therapy and serum albumin and cholesterol. Esophageal fistula parameters include fistula type and therapy of fistula. The details are given in **Table 1**.

**Table 1:** Characteristics of patients.

	Characteristics	Training set		Validation set	
		Case group	Control group	Case group	Control group
		(n = 130) (%)	(n = 260) (%)	(n = 56) (%)	(n = 112) (%)
**1. General parameters**	**Age (years)**				
	**<**60	54 (40.8)	76 (29.2)	30 (53.6)	26 (23.2)
	≥60	76 (59.2)	184 (70.8)	26 (46.4)	86 (76.8)
	**ECOG PS**				
	0	51 (39.2)	163 (62.7)	18 (32.1)	67 (59.8)
	1	63 (48.5)	83 (31.9)	32 (57.1)	41 (36.6)
	2	10 (7.7)	12 (4.6)	3 (5.4)	4 (3.6)
	3	6 (4.6)	2 (0.8)	3 (5.4)	0
	**BMI (kg/m²)**				
	<18.5	18 (13.8)	31 (11.9)	8 (14.3)	9 (8.0)
	18.5-23.9	75 (57.7)	143 (55.0)	33 (58.9)	64 (57.1)
	24-27.9	30 (23.1)	63 (24.2)	14 (25.0)	29 (25.9)
	≥28	7 (5.4)	23 (8.8)	1 (1.8)	10 (8.9)
	**History of Smoking**				
	yes	81 (62.3)	164 (63.1)	36 (64.3)	68 (60.7)
	no	49 (37.7)	96 (36.9)	20 (35.7)	44 (39.3)
	**history of drinking**				
	yes	74 (56.9)	130 (50.0)	36 (64.3)	57 (50.9)
	no	56 (43.1)	130 (50.0)	20 (35.7)	55 (49.1)
	**History of hypertension**				
	yes	29 (22.3)	64 (24.6)	13 (23.2)	30 (26.8)
	no	101 (77.7)	196 (75.4)	43 (76.8)	82 (73.2)
	**History of diabetes**				
	yes	12 (9.2)	23 (8.8)	3 (5.4)	5 (4.5)
	no	118 (90.8)	237 (91.2)	53 (95.6)	107 (95.5)
	**History of coronary heart disease**				
	yes	4 (3.1)	19 (7.3)	2 (3.6)	7 (6.3)
	no	126 (96.9)	241 (92.7)	54 (96.4)	105 (93.8)
	**Eating obstruction**				
	yes	111 (85.4)	224 (86.2)	52 (92.9)	92 (82.1)
	no	19 (14.6)	36 (13.8)	4 (7.1)	20 (17.9)
	**Serum albumin (g/L)**				
	≥35	114 (87.7)	253 (97.3)	48 (85.7)	110 (98.2)
	<35	16 (12.3)	7 (2.7)	8 (14.3)	2 (1.8)
	**Serum cholesterol (mmol/L)**				
	≥4.40	79 (60.8)	178 (68.5)	38 (67.9)	76 (67.9)
	<4.40	51 (39.2)	82 (31.5)	18 (32.1)	36 (32.1)
**2. Diagnostic parameters**	**T stage**				
	T1-3	84 (64.6)	225 (86.5)	35 (62.5)	92 (82.1)
	T4	46 (35.4)	35 (13.5)	21 (37.5)	20 (17.9)
	**N stage**				
	N0-1	59 (45.4)	155 (59.6)	15 (26.8)	72 (64.3)
	N2-3	71 (54.6)	105 (40.4)	41 (73.2)	40 (35.7)
	**Stage**				
	I stage	0	2 (0.8)	0	1 (0.9)
	II stage	10 (7.7)	36 (13.8)	2 (3.6)	18 (16.1)
	III stage	75 (57.7)	149 (57.3)	24 (42.9)	61 (54.5)
	IV stage	45 (34.6)	73 (28.1)	30 (53.6)	32 (28.6)
	**Tumor site**				
	proximal esophagus	40 (30.8)	70 (26.9)	15 (26.8)	21 (18.8)
	middle esophagus	44 (33.8)	74 (28.5)	20 (35.7)	38 (33.9)
	distal esophagus	46 (35.4)	116 (44.6)	21 (37.5)	53 (47.3)
	**Longitudinal length of lesions(cm), mean ± SD**	6.64 ± 2.41	5.93 ± 3.23	7.23 ± 2.99	5.41 ± 2.64
	**Pathological type**				
	squamous carcinoma	125 (96.2)	240 (92.3)	52 (92.9)	103 (92.0)
	adenocarcinoma	1 (0.8)	9 (3.5)	1 (1.8)	3 (2.7)
	neuroendocrine carcinoma	3 (2.3)	9 (3.5)	3 (5.4)	3 (2.7)
	adenosquamous carcinoma	1 (0.8)	2 (0.8)	0	3 (2.7)
	**General type**				
	medullary type	51 (39.2)	142 (54.6)	26 (46.4)	58 (51.8)
	mushroom type	34 (26.2)	46 (17.7)	8 (14.3)	25 (22.3)
	ulcerative type	36 (27.7)	55 (21.2)	11 (19.6)	19 (17.0)
	constrictive type	7 (5.4)	10 (3.8)	8 (14.3)	5 (4.5)
	cavity type	2 (1.5)	7 (2.7)	3 (5.4)	5 (4.5)
**3. Therapeutic parameters**	**Chemotherapy**				
	yes	91 (70.0)	229 (88.1)	45 (80.4)	93 (83.0)
	no	39 (30.0)	31 (11.9)	11 (19.6)	19 (17.0)
	**Taxol chemotherapy**				
	yes	76 (58.5)	175 (67.3)	35 (62.5)	65 (58.0)
	no	54 (41.5)	85 (32.7)	21 (37.5)	47 (42.0)
	**Chemotherapy**				
	0 line	39 (30.0)	31 (11.9)	11 (19.6)	19 (17.0)
	1 line	69 (53.1)	168 (64.6)	31 (55.4)	75 (67.0)
	2 line	19 (14.6)	46 (17.7)	11 (19.6)	15 (13.4)
	3 line and more	3 (2.3)	15 (5.8)	3 (5.4)	3 (2.7)
	**Radiotherapy**				
	yes	74 (56.9)	177 (68.1)	29 (51.8)	82 (73.2)
	no	56 (43.1)	83 (31.9)	27 (48.2)	30 (26.8)
	**Concurrent radiochemotherapy**				
	yes	17 (13.1)	47 (18.1)	6 (10.7)	25 (22.3)
	no	113 (86.9)	213 (81.9)	50 (89.3)	87 (77.7)
	**Re-radiotherapy**				
	yes	3 (2.3)	2 (0.8)	3 (5.4)	2 (1.8)
	no	127 (97.7)	258 (99.2)	53 (94.6)	110 (98.2)
	**Fraction of radiation (patients who received radiotherapy)**				
	≤30	58 (78.4)	126 (71.2)	22 (75.9)	54 (65.9)
	>30	16 (21.6)	51 (28.8)	7 (24.1)	28 (34.1)
	**Total dose (patients who finished the radiotherapy)**				
	≥60Gy	25 (33.8)	91 (51.4)	9 (31.0)	48 (58.5)
	≥50 <60Gy	29 (39.2)	64 (36.2)	14 (48.3)	23 (28.0)
	<50Gy	20 (27.0)	22 (12.4)	6 (20.7)	11 (13.4)
	**Average single dose (patients who received radiotherapy)**				
	≤1.8	29 (39.2)	56 (31.3)	9 (31.0)	24 (29.3)
	>1.8	45 (60.8)	121 (68.4)	20 (69.0)	58 (70.7)
	**Radiotherapy technology (patients who received radiotherapy)**				
	general radiotherapy	1 (1.4)	0	0	0
	3DCRT	24 (32.4)	38 (21.5)	8 (27.6)	12 (14.6)
	IMRT	49 (66.2)	139 (78.5)	21 (72.4)	69 (85.3)
	**Radiotherapy range (esophagus)**				
	yes	73 (56.2)	175 (67.3)	29 (51.8)	82 (73.2)
	no	57 (43.8)	85 (32.7)	27 (48.2)	30 (26.8)
	**Radiotherapy range (metastatic lymph nodes)**				
	yes	58 (44.6)	144 (55.4)	22 (39.3)	57 (50.9)
	no	72 (55.4)	116 (44.6)	34 (60.7)	55 (49.1)
	**Radiotherapy area (lymphatic drainage area)**				
	yes	40 (30.8)	101 (38.8)	15 (26.8)	45 (40.2)
	no	90 (69.2)	159 (61.2)	41 (73.2)	67 (59.8)
	**Target therapy**				
	yes	8 (6.2)	17 (6.5)	4 (7.1)	9 (8.0)
	no	122 (93.8)	243 (93.5)	52 (92.9)	103 (92.0)

### Image Acquisition

All patients underwent esophagoscopy, esophagogram and contrast-enhanced CT scan of neck, chest, and abdomen before treatment. We collected pre-treatment CT imaging and diagnostic CT of esophageal fistula. Intravenous contrast enhancement was used for all patients. The CT-scans were acquired by SOMATOM Definition AS (Siemens Healthineers) using a tube voltage of 120 kVp, a tube current of 200 mAs, a detector of 64×0.625 mm and a beam pitch of 1.5. Esophageal tumor boundaries on all 558 pre-treatment CT imaging were manually delineated with reference to esophagoscopy, barium meal or PET-CT in mediastinal window twice using 3D-Slicer by two experienced radiologists separately to reduce the deviation. For patients with satellite tumors, only the primary tumor or the tumor that caused esophageal fistula was appreciated.

### Deep Learning Neural Network

**Figure 1:**
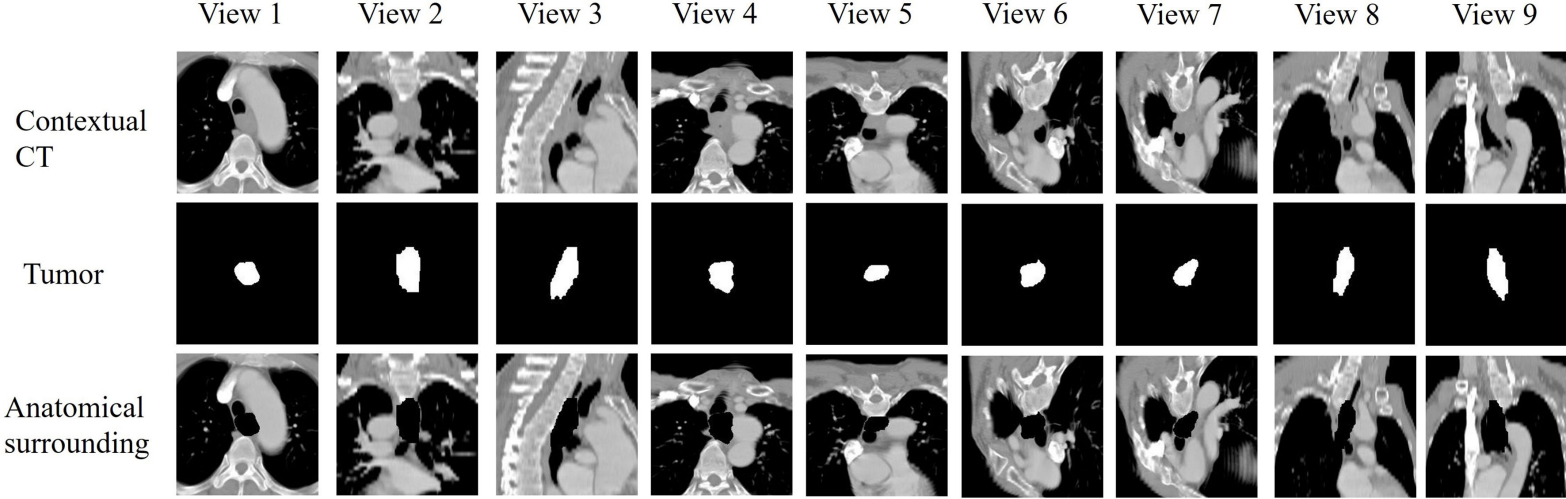
The nine views of planes we extracted. The nine views included transverse, sagittal, coronal and six diagonal planes.

To extract radiographic features from CT, we developed an attentional multi-view multi-scale CNN model (AMM-CNN). The inputs of the network were nine views of panels where there are patches of contextual CT, segmented tumor and neighboring structures in each view. To extract nine views and patches, CT images were firstly resampled to a voxel size of 1×1×1 mm^3^. A 200×200×200 mm^3^ cube was defined as located at the center of manually segmented tumor volume. We used its transverse, sagittal, coronal and six diagonal planes as nine views (**Figure 1**). The contextual patch was defined as a 2D slice in a view from the CT cube, which represented the contextual information of the tumor and its neighboring environment. The tumor patch was extracted from the cube of the segmented tumor volume, providing an explicit shape of tumor and boundary information. To generate anatomical surrounding patch, the pixels inside the tumor were set as zero on contextual patch.

Clinical records were fed into a neural network for high-level representation extraction. Finally, the radiographic features and clinal factor representation are associated with a fully connected layer for patient-level risk prediction using SoftMax classifier.

### Performance Evaluation

The performance of the proposed risk prediction model was validated by comparing it with the risk prediction model using CT images alone and clinical records data alone.

Evaluation measures included C-index, sensitivity, and specificity. Given true positive (TP), false negative (FN), true negative (TN), and false positive (FP) numbers, sensitivity and specificity are obtained as *sensitivity* = *TP*/ (*TP*+*FN*), *specificity* = *TN*/ (*TN*+*FP*).

## Results

### Patient Characteristics

**Figure 2:**
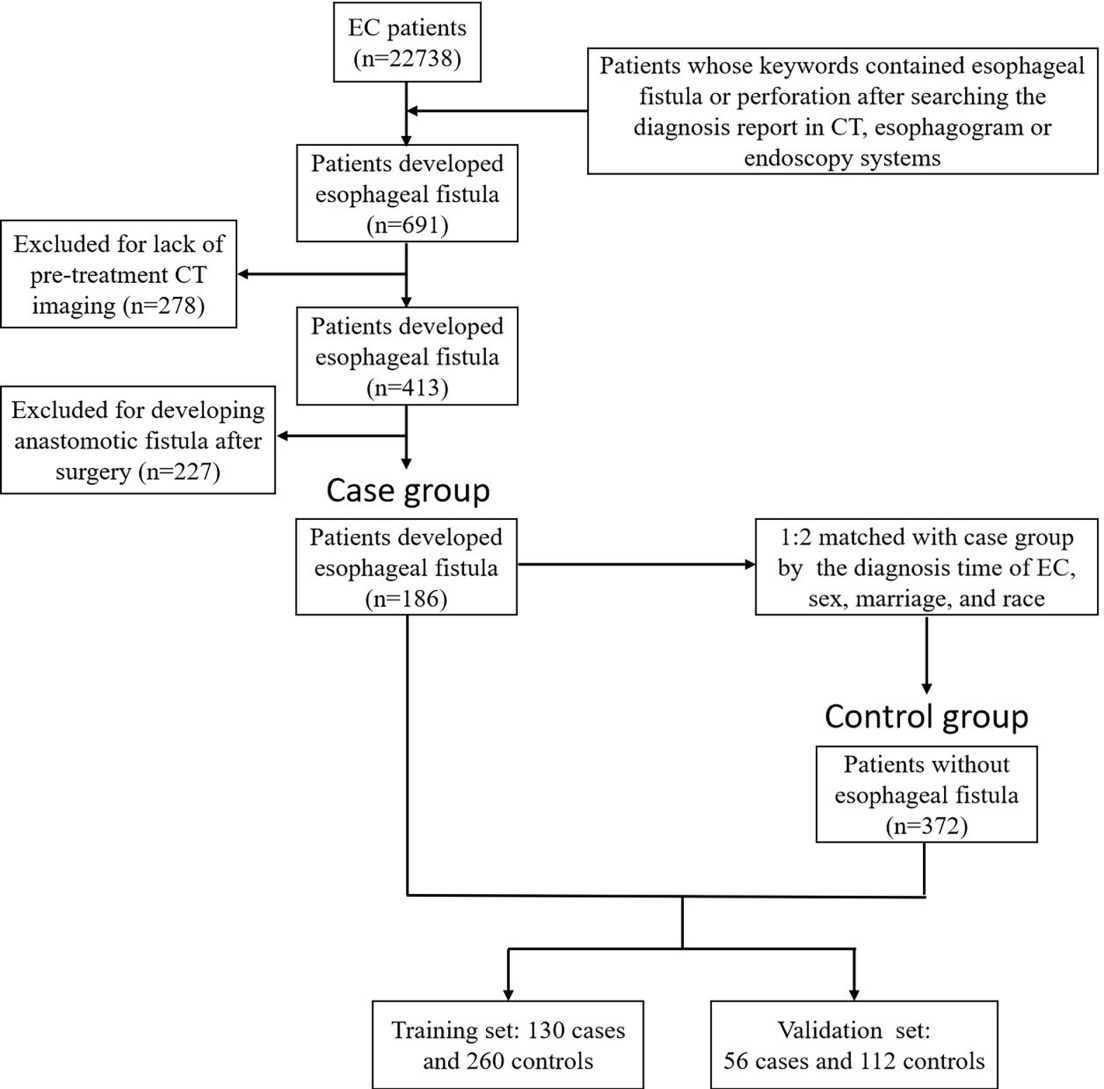
The overall workflow of patients. We retrospectively screened 22738 patients, and finally 186 were enrolled in the case group and 372 in the control group. All patients were randomly divided into 70% (training set) and 30% (validation set). Key words esophageal fistula or perforation, and esophageal cancer were set in the imaging system. After excluding duplicate patients, a total of 691 patients with esophageal fistula were collected. Then, patients with lack of diagnostic CT (n=278) and with postoperative anastomotic leakage (n=227) were excluded. Finally, 186 esophageal fistula patients were enrolled.

691 patients developed esophageal fistula during the study period. 413 had complete pre-treatment CT imaging. All perforations were developed at the location where the tumor invaded the esophagus. After excluding 227 patients with postoperative anastomotic fistulas who had surgical operations, 186 patients were finally enrolled in the case group. 372 controls never received esophageal operation matching the case cases. The detailed workflow is given in **Figure 2**. During the cross-validation process, in each round, all the patients were divided into a training set (randomly selected 130 pairs of positive patients and the controls) and a testing set (remaining 56 pairs of positive and controls) randomly.

Among all 558 eligible patients, 468 (83.9%) are male and 90 (16.1%) are female. The median age is 61 (range 41-85) in the case group and 64 (range 37-89) in the control group separately. Patients with squamous carcinoma predominated account for 93.2% where most of them had stage III EC (52.2%) with T3 (63.6%) or N1 (40.1%) disease. Before developing perforation, the proportions of patients who received chemotherapy or radiotherapy were 71.5% and 54.3% respectively, while 45.7% of patients received both of them, and 12.4% received concurrent chemoradiotherapy. Besides, 37 (19.9%) patients developed esophageal fistula before treatment. The median interval time from baseline CT to the diagnosis of esophageal fistula was 5 days (3-9 days). The interval time between the development of esophageal fistula and the diagnosis of esophageal cancer ranged from 3 to 1401 days with a median value of 72 days. The median survival time after esophageal fistula is 2.9 months.

In the case group, 90 patients (48.4%) had fistula formation to the trachea or bronchus, 91 patients (48.9%) had fistula formation to the mediastinum, and two patients (1.1%) and one patient (0.5%) had fistula formation to the pleural cavity and the arteria, respectively. Two patients developed two kinds of fistula simultaneously. After the development of fistula, most patients received nutritional support. Meanwhile, some of the patients accepted nutrient canal (34.9%), esophageal stent (31.7%), gastrostomy (7.5%), and radical resection (0.5%). Conservative treatment represents only intravenous nutrition, without nutrition tubes or gastrostomy. Of all 558 patients, no patient was placed with stent before treatment or received intraluminal radiotherapy. The esophageal fistula characteristics are listed in **Table 2**.

**Table 2:** Esophageal fistula characteristics of the patients.

Characteristics	Esophageal fistula	
	Training set	Validation set
	(n = 130) (%)	(n = 56) (%)
**Treatment before fistula**		
chemotherapy	88 (67.7)	45 (80.4)
radiotherapy	73 (56.2)	28 (50.0)
radiochemotherapy	60 (46.2)	25 (44.6)
concurrent radiochemotherapy	17 (13.1)	6 (10.7)
target therapy	1 (0.8)	1 (1.8)
none	29 (22.3)	8 (14.3)
**Fistula type**		
esophageal- respiratory	62 (47.7)	27 (48.2)
esophageal- mediastinum	64 (49.2)	27 (48.2)
esophageal- pleural fistula	1 (0.8)	1 (1.8)
esophageal- vascular fistula	1 (0.8)	0
both esophageal- respiratory and esophageal- mediastinum fistula	1 (0.8)	0
both esophageal- mediastinum and esophageal- vascular fistula	1 (0.8)	0
**Therapy of fistula**		
nutrient canal	47 (36.2)	18 (32.1)
esophageal stent	44 (33.8)	15 (26.8)
conservative treatment	30 (23.1)	17 (30.4)
gastrostomy	8 (6.2)	6 (10.7)
radical resection	1 (0.8)	0

### Correlation Between Clinical Data and the Esophageal Fistula

In univariate logistic regression analysis, there are significant differences between patients with and without fistula in age, ECOG PS score, serum albumin, T4 stage, N stage, stage, longitudinal length of lesions, general type, and treatment-related parameters. All significant factors were further included in the multiple regression analysis. Age, ECOG PS score, serum albumin, T4 stage, N stage, general type, chemotherapy, total dose of radiotherapy, and radiotherapy range (metastatic lymph nodes) are independent risk factors for esophageal fistula. The detailed results are shown in **Table 3**.

**Table 3:** Univariate and multivariate logistic regression analysis of clinical characteristics in the training set.

	Characteristics	Univariate		Multivariate	
		p	OR (95.0% CI)	p	OR (95.0% CI)
**1. General parameters**	**Age**	0.007	0.97 (0.95-0.99)	<0.001	0.91 (0.88-0.95)
	**ECOG PS**				
	0	<0.001	1.00 (reference)	0.001	1.00 (reference)
	1		2.36 (1.48-3.76)		4.01 (2.04-7.90)
	2		2.73 (1.09-6.84)		2.92 (0.78-10.89)
	3		8.03 (1.59-40.68)		2.20 (0.26-18.51)
	**BMI (kg/m²)**				
	<18.5	0.642	1.00 (reference)		
	18.5-23.9		0.89 (0.46-1.70)		
	24-27.9		0.82 (0.39-1.69)		
	≥28		0.53 (0.19-1.48)		
	**History of Smoking**				
	no	0.869	0.96 (0.59-1.56)		
	yes				
	**history of drinking**				
	no	0.135	1.46 (0.89-2.41)		
	yes				
	**History of hypertension**				
	no	0.620	0.88 (0.54-1.45)		
	yes				
	**History of diabetes**				
	no	0.903	1.05 (0.52-2.12)		
	yes				
	**History of coronary heart disease**				
	no	0.100	0.39 (0.13-1.20)		
	yes				
	**Eating obstruction**				
	Grade 0	0.751	1.00 (reference)		
	Grade 1		0.82 (0.49-1.38)		
	Grade 2		1.08 (0.61-1.91)		
	Grade 3		1.38 (0.75-2.53)		
	Grade 4		–		
	**Serum albumin (g/l)**				
	<35	<0.001	0.89 (0.85-0.94)	0.001	0.88 (0.82-0.95)
	≥35				
	**Serum cholesterol (mmol/l)**				
	<4.40	0.141	0.83 (0.66-1.06)		
	≥4.40				
**2. Diagnostic parameters**	**T stage**				
	T1-3	<0.001	3.76 (2.17-6.51)	<0.001	5.08 (2.27-11.41)
	T4				
	**N stage**				
	N0-1	0.008	1.82 (1.17-2.82)	0.006	2.58 (1.31-5.10)
	N2-3				
	**Stage**				
	I-II	0.057	2.03 (0.98-4.19)	0.586	1.37 (0.44-4.25)
	III-IV				
	**Tumor site**				
	proximal esophagus	0.184	1.00 (reference)		
	middle esophagus		1.05 (0.61-1.82)		
	distal esophagus		0.67 (0.39-1.14)		
	**Longitudinal length of lesions**	0.030	1.09 (1.01-1.17)	0.248	1.07 (0.95-1.21)
	**Pathological type**				
	squamous carcinoma	0.492	1.00 (reference)		
	adenocarcinoma		0.22 (0.03-1.75)		
	neuroendocrine carcinoma		0.67 (0.18-2.47)		
	adenosquamous carcinoma		0.94 (0.08-10.45)		
	**General type**				
	medullary type	0.055	1.00 (reference)	0.042	1.00 (reference)
	mushroom type		2.04 (1.18-3.53)		3.06 (1.32-7.08)
	ulcerative type		1.80 (1.06-3.06)		1.95 (0.93-4.10)
	constrictive type		1.92 (0.71-5.23)		1.59 (0.40-6.30)
	cavity type		0.72 (0.15-3.53)		0.35 (0.05-2.55)
**3. Therapeutic parameters**	**Chemotherapy**				
	no	<0.001	0.33 (0.20-0.55)	0.020	0.28 (0.10-0.82)
	yes				
	**Taxol chemotherapy**				
	no	0.064	0.64 (0.40-1.03)	0. 428	0.68 (0.26-1.78)
	yes				
	Chemotherapy*				
	0 line	0.001	1.00 (reference)		
	1 line		0.35 (0.20-0.60)		
	2 line		0.32 (0.15-0.68)		
	3 line and more		0.17 (0.05-0.65)		
	**Radiotherapy**				
	no	0.030	0.61 (0.39-0.95)	0.519	6.69 (0.02-2147.90)
	yes				
	**Concurrent radiochemotherapy**				
	no	0.205	0.67 (0.37-1.24)		
	yes				
	**Re-radiotherapy**				
	no	0.229	3.00 (0.50-17.95)		
	yes				
	**Fraction of radiation (all patients)**				
	≤30	0.086	0.60 (0.33-1.08)	0.255	0.57 (0.22-1.50)
	>30				
	**Total dose (all patients)**	0.002	0.99 (0.98-0.99)	0.037	0.97 (0.93-0.99)
	**Average single dose (all patients)**	0.024	0.77 (0.61-0.97)	0.473	0.54 (0.10-2.88)
	Radiotherapy technology (all patients)*				
	none	0.092	1.00 (reference)		
	general radiotherapy		0.52 (0.32-0.85)		
	3DCRT		0.98 (0.51-1.86)		
	IMRT		136939.79 (0.00 -2.158E + 262)		
	TOMO		0.58 (0.06-5.60)		
	**Radiotherapy range (esophagus)**				
	no	0.030	0.61 (0.39-0.95)	0.291	11.23 (0.13-1004.09)
	yes				
	**Radiotherapy range (metastatic lymph nodes)**				
	no	0.043	0.64 (0.41-0.99)	0.012	0.23 (0.07-0.73)
	yes				
	**Radiotherapy area (lymphatic drainage area)**				
	no	0.111	0.69 (0.44-1.09)		
	yes				
	**Target therapy**				
	no	0.885	0.94 (0.40-2.21)		
	yes				

^*^Due to duplication, these two characteristics were not included in multivariate analysis.

### Deep Learning Prediction Model Implementation

**Figure 3:**
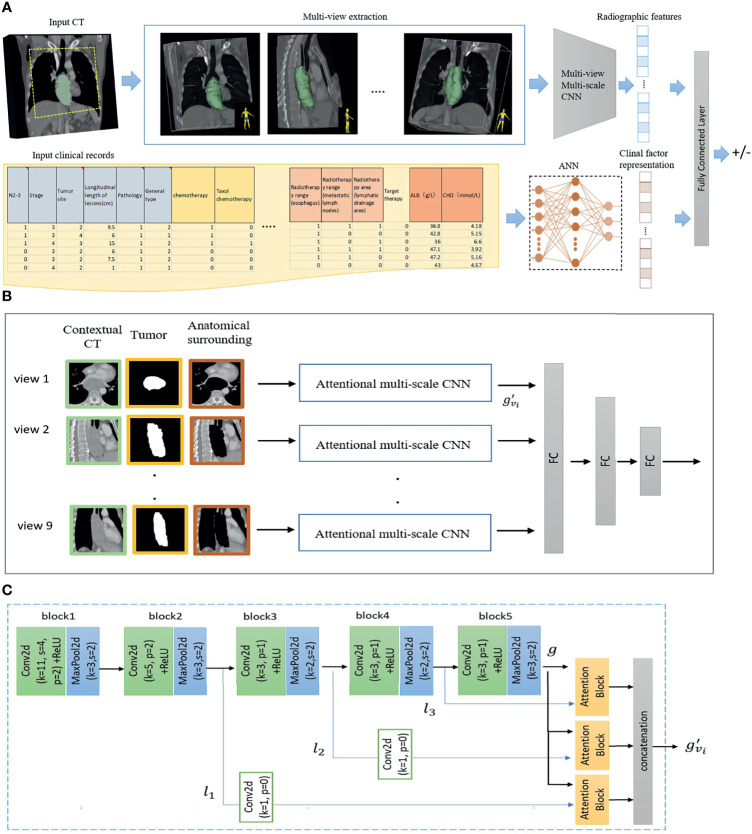
The proposed risk prediction model **(A)** Given an input CT and segmented tumor, 9 views of planes are extracted. Contextual CT, tumor and anatomical surrounding patches of each view are sent to a Multi-view Multi-scale CNN model for radiographic feature extraction. For the input clinical records, an artificial neural network (ANN) is used to extract clinical factor representations. Finally, radiographic and clinical features are fused by a fully connected layer for esophageal fistula prediction. Architecture of Multi-view Multi-scale CNN is given in **(B)**. Contextual CT, tumor and anatomical surrounding patches extracted from each view are sent to **(C)** attentional multi-scale CNN. Multi-scale features are extracted from the second, third and fourth blocks in the CNN, and adaptively fused by attention blocks.

The detailed architecture of AMM-CNN is given in **Figure 3**. AMM-CNN adopts the architecture of AlexNet (13) for image feature extraction and has an attentional fusion module to adaptively integrate multi-view multi-level image features. Given contextual CT, tumors, and surrounding tissues from 9 views, AMM-CNN generates 512 radiographic features. 20 clinical representations were learnt by the NN from input clinical records.

To improve the learning effectiveness, data augmentation was performed, including pixel shifting and rotation for the training set. As there were imbalanced positive and negative cases, shifting operations of -10, -5, 0, + 5, +10 pixels along the x and y-axis and rotations of -10, +10 degrees were performed for positive cases, resulting in 9750 positive samples. For negative training cases, 9360 negative samples were obtained after shifting operations of -5, 0, + 5, +10 along x and -5, 0, + 5 along y-axis, and rotations of -10, +10 degrees.

Combining clinical features and CT imaging, deep learning achieved a C-index of 0.921 in the internal validation and 0.901 in the external validation, which outperformed CT imaging alone (internal validation: 0.902; external validation: 0.857) and clinical data alone (internal validation: 0.855; external validation: 0.780). The sensitivity was 0.835, and specificity was 0.918. The integrative model produced higher predictive performance than models using single modality data. For the clinical characteristics, the C-index obtained by deep learning is 0.780, which is better than the traditional logistics regression model (internal validation: 0.823, external validation: 0.734).

### Interpretability of the Model

**Figure 4:**
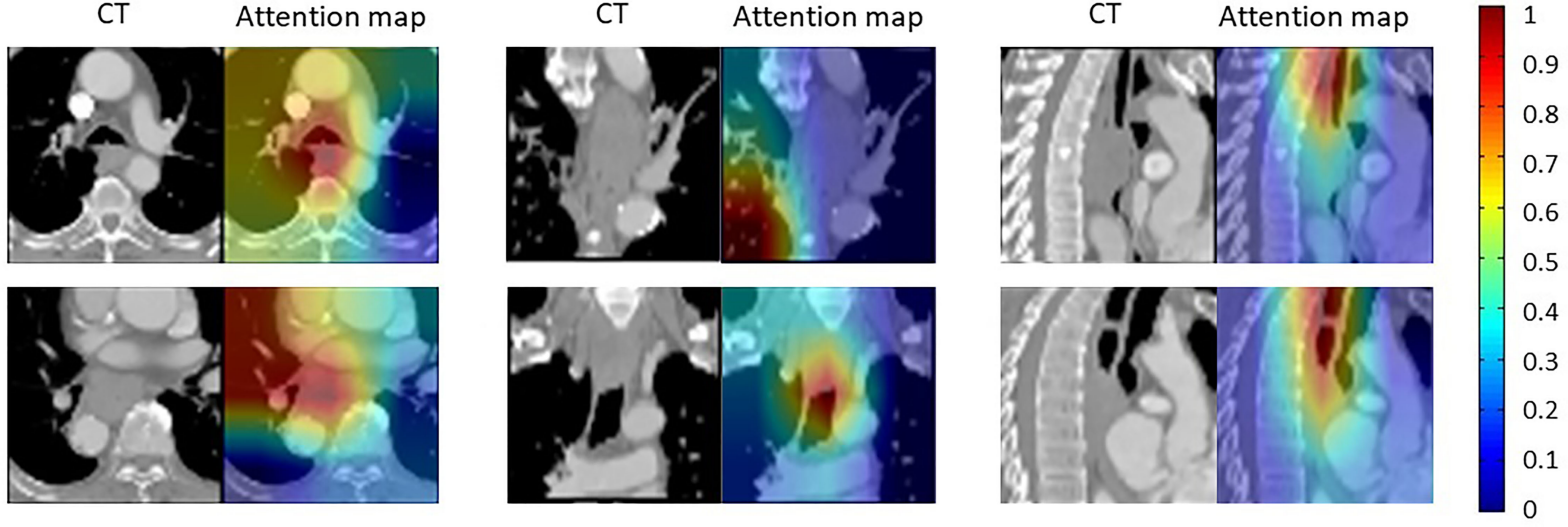
Visualization of attention maps of six patient examples. The attention heatmap shows the risk model’s focus on esophageal fistula prediction. The hotter areas indicate that the tissues have greater impact on esophageal fistula formation. As shown, the tumor boundaries and the hypoechoic area inside the tumor are more concerned.

To study the interpretability of the model, we draw the attention map to explain the focus of the neural network on CT images. As shown in **Figure 4**, hotter areas of the attention map represent the tissues predicted by the algorithm that has a higher impact on the formation of esophageal fistula. Our results show that there were usually two locations that receive more attention. One is the border of the tumor, and the other is the hypoechoic area inside the tumor. The visual interpretation further proved the effectiveness of the model.

## Discussion

Esophageal fistula is a fatal complication of EC. Therefore, a risk prediction model integrating CT imaging and clinical features is worth investigation. In this study, we used the deep learning method to comprehensively analyze the influence of various parameters on the esophageal fistula, including clinical parameters such as stage, treatment, and CT imaging. Deep learning models can directly learn patient characteristics from raw data or imaging without feature selection or design (14). Therefore, more complete data can be included for analysis. To our knowledge, this is the first deep learning model that uses different types of parameters for esophageal fistula prediction.

The prediction performance of integrative deep learning model is better than that of a single parameter model (C-index: 0.901 *vs* 0.857, 0.780). Because deep learning algorithms can integrate clinical parameters and CT images well. Deep learning is very suitable for the analysis of multi-domain parameters, such as the fusion of histopathological images and genomic data (15). The integrative model contains more information than a single model and can achieve better prediction performance.

Deep learning model is also superior than traditional logistics regression. The first reason is that intuitive tumor information can be obtained from CT imaging, including the tumor size, density and invasion degree of surrounding tissues, which are all related to the esophageal fistula. The second reason is that the nomogram was established in previous studies to predict esophageal fistula (16). However, the nomogram was developed based on logistics regression analysis, which couldn’t capture the nonlinear relationship between risk factors and esophageal fistula, and the number of risk factors included was relatively small. Therefore, the performance of this nomogram is limited. Our prediction model provides an end-to-end data-driven trainable approach to learn the mapping from input images to output risk grades. The mapping serves as a feature extractor, which is automatically learned during the training process. As a result, the extractor is more general and adjustable when compared with explicitly defined hand-crafted features in previous research (17). In addition, a large volume of training data and deep learning technique equips our model the ability to extract more in-depth features and underlying image information. Therefore, deep learning models are expected to replace logistics regression analysis.

Deep learning has a certain interpretability for the image analysis (18). This study shows that the tumor boundaries and the hypoechoic area inside the tumor have the greatest predictive significance for esophageal fistula. The tumor boundaries are adjacent to the normal tissue, which can represent the status of tumor invasion. The hypoechoic area inside the tumor is related to the tumor growth rate and malignancy. This proves that our model is reasonable.

Clinicians can use this model to evaluate esophageal fistula risk before or during treatment. For high-risk patients, the dose of chemotherapy or radiotherapy can be appropriately reduced with enhanced nutritional support. In addition, the examination should also be taken more frequently. Although it is generally believed that one of the adverse reactions of radiotherapy is esophageal fistula, some studies believe that radiotherapy can promote the healing of esophageal fistula, and further research on the frequency and dose of radiotherapy is needed.

This study has several limitations. First, deep learning has poor interpretability of clinical parameters, and it is difficult to analyze which clinical parameters have a greater impact on the esophageal fistula. Second, the study is a single-center study. Data from other regions and centers are required for further validation.

## Conclusion

In this study, we developed a deep learning model to integrate CT imaging and clinical information for esophageal fistula prediction in EC patients. We suggest this study and the developed model can facilitate individualized treatment, leading to maximized therapeutic gain.

## Data Availability Statement

The raw data supporting the conclusions of this article will be made available by the authors, without undue reservation.

## Ethics Statement

The studies involving human participants were reviewed and approved by Institutional Review Board of Shandong Cancer Hospital and Institute, Shandong First Medical University, and Shandong Academy of Medical Sciences. The ethics committee waived the requirement of written informed consent for participation.

## Author Contributions

YX, TD, and LW contributed to the conception and design of the manuscript. BZ, BF, and WL collected the data and CT images. YX and HC performed data analysis and drafted the manuscript. SW, XS, and JY revised the original manuscript. All authors contributed to the article and approved the submitted version.

## Funding

This study was funded by the following grant: Natural Science Foundation of Shandong Province (Grant No. ZR2019LZL012), the Innovation Project of Shandong Academy of Medical Sciences (2019-04), and the Academic Promotion Program of Shandong First Medical University (2019ZL002).

## Conflict of Interest

The authors declare that the research was conducted in the absence of any commercial or financial relationships that could be construed as a potential conflict of interest.

## Publisher’s Note

All claims expressed in this article are solely those of the authors and do not necessarily represent those of their affiliated organizations, or those of the publisher, the editors and the reviewers. Any product that may be evaluated in this article, or claim that may be made by its manufacturer, is not guaranteed or endorsed by the publisher.
